# The effect of dental loupes on postural ergonomics during non-surgical periodontal therapy

**DOI:** 10.1038/s41598-025-28124-0

**Published:** 2025-12-24

**Authors:** Resül Çolak, İsmail Gül, Merve Küçükoğlu Çolak

**Affiliations:** Department of Periodontics, Faculty of Dentistry, Zonguldak Bülent Ecevit University, 67600 Esenköy/Kozlu, Zonguldak, Turkey

**Keywords:** Dental loupes, Ergonomics in dentistry, Musculoskeletal disorders, Postural assessment, Health care, Medical research, Risk factors, Materials science

## Abstract

To evaluate the effect of prismatic dental loupes on clinician posture during periodontal treatment performed by periodontology assistants. This cross-sectional observational study was conducted using a counterbalanced crossover design. Ten periodontology assistants performed supragingival scaling on two different patients; once with and once without loupes. Posture was assessed using Branson’s Posture Assessment Instrument (BPAI) through photographic evaluation at the 1st, 3rd, and 5th minutes of treatment. BPAI scores were compared between the two conditions, and correlations among posture components were analyzed. The use of loupes significantly improved overall posture, with lower total BPAI scores in the loupe condition. Significant improvements were observed in the hip, trunk, head/neck, and shoulder regions, while wrist posture did not differ significantly. A strong positive correlation was found between total BPAI and head/neck scores, indicating the influence of head/neck alignment on overall posture. Magnification loupes contribute to improved clinician posture, especially in the head/neck region, which strongly correlated with overall posture. The observed interdependence among body regions supports the need for holistic posture assessment. These findings highlight the ergonomic benefits of loupes in reducing musculoskeletal risks in dental practice. Incorporating magnification loupes into clinical practice may improve operator posture and contribute to long-term occupational health among dental professionals.

## Introduction

Musculoskeletal disorders (MSDs) are among the most common occupational disorders encountered by dentists and can even begin during university education^1–3^. Dentists and dental hygienists often adopt awkward and potentially harmful postures, such as leaning their head forward, hunching their shoulders, or stretching their bodies beyond a stable position^3–6^. It has been reported that muscles and joints are strained because of impaired posture, which triggers symptoms such as back pain, headache, and neck and shoulder pain^7^. According to De Sio et al., the prevalence of work-related pain among dentists ranges from 54 to 93%^8^. This is supported by further research showing that approximately 68% of dentists report musculoskeletal discomfort in the neck, back, and shoulder regions^9^.

Postural distortions have been reported as a consequence of working in confined treatment spaces and maintaining static positions to enhance visual access^10^. Over time, these compromised working conditions may contribute to the development or worsening of musculoskeletal disorders, especially when combined with poor postural habits^11^. This may lead to increased physical limitations and reduced work capacity, ultimately resulting in negative consequences, both economically and with regard to the quality of life^12,13^. However, this risk can be reduced through ergonomic strategies that promote and/or maintain a neutral posture^12,13^.

Ergonomics is a scientific discipline that aims to maximize the safety and efficiency of practitioners, while minimizing pain and fatigue. This involves developing techniques, designing equipment, and organizing workspaces^14^. To reduce the risk of MSDs within the dental profession, a combination of proper posture training, improvement in dental equipment, and interventions aimed at correcting posture is recommended^15^.

Cervera-Espert et al. reported that only 28.6% of dentists maintained acceptable and uncompromising postures. Although ergonomic principles are well understood in theory by most dentists, their practical implementation in everyday clinical settings remains limited^16^. Additionally, a systematic review of 41 studies published between 2005 and 2017 reported that the annual prevalence of MSDs among dentists in Western countries reached 78%^17^. This high prevalence of MSDs clearly demonstrates the importance of improving ergonomic conditions during occupation. The discrepancy between the theory and practice of ergonomic concepts can be minimized through postural modifications using loupes. Studies involving dental students have shown that using loupes improves neck posture, considering cervical angular deviation, both during preclinical training and clinical procedures such as periodontal probing and diagnosis^18–20^.

However, while working with loupes reduces cervical angular deviation, the angle still exceeds the range considered neutral. According to Rucker et al.^21^, this may be due to the need for precise adjustment of the declination angle of the loupe lenses to achieve a balance between visual strain and neck posture. A forward head posture creates torque in the neck, increasing muscular load and making it more sensitive to additional head-borne weight, such as loupes or headlamps^22^.

Adjusting the angle of prismatic loupes can influence head tilt and consequently reduce this torque. While prismatic loupes may have the potential to reduce physical workload in clinicians, there is no current consensus in the literature regarding the effects of different loupe angles. Therefore, further studies are recommended to evaluate the feasibility and effectiveness of prismatic loupes^23^.

The literature suggests that the use of dental loupes when performing dental treatments may improve the quality of treatment and has the potential to reduce the likelihood of MSDs; however, indications of a physiological benefit are anecdotal^24–28^. Although the use of loupes is recommended in dentistry, there are few evidence-based studies on the effects of loupes on working posture, and the majority of studies are limited to undergraduate dental students and hygienists with limited professional experience^4,29,30^. To the best of our knowledge, no study has evaluated the effect of using dental loupes use on the posture of periodontology research assistants.

This observational comparative study aimed to evaluate the effect of using prismatic loupes on the posture of periodontology research assistants during periodontal treatment. The H1 hypothesis regarding the primary outcome of the study is that there is a difference in periodontology research assistants posture in loupe users (test) compared to the group not using the loupe (control).

## Materials and methods

### Study design and ethical approval

The study was designed as a cross-sectional single-centre observational study. Prior to starting, ethical approval was obtained from the Zonguldak Bülent Ecevit University Non-Interventional Clinical Research Ethics Committee, dated 04.12.2024 and numbered 2024/27. In accordance with the Declaration of Helsinki, written informed consent was obtained from all periodontology research assistants and patients participating in our study. All participants of this article provided the consent to publish.

### Sample and criteria

The sample size of the study was determined based on the differences observed in the subcomponents of the BPAI total score between the study groups. The power analysis was performed using the G*Power software (version 3.1.9.7, Franz Faul, University of Kiel, Kiel, Germany), referencing the study by Bud et al.^31^. According to this reference, a mean difference of 0.35 ± 0.06 was considered statistically significant, and the corresponding effect size was calculated to be 1.20.

Assuming a 95% confidence level (α = 0.05) and a power of 80% (1–β = 0.80), the power analysis revealed that a minimum of 8 participants would be required to detect a significant difference between the groups (non-centrality parameter λ = 3.595253, critical t = 1.85955, df = 6). To meet this requirement and ensure sufficient statistical power, the study was completed with a total of 10 participants.

### Specific inclusion criteria

-  ≥ 18 years old,
- Systemically healthy (e.g., diabetes, rheumatologic or neuromuscular disorders),
- Graduated from dental school and specialised in periodontology,
- No eye problems (no use of contact lenses or prescription glasses),
- No medical history of diagnosed spinal or cervical joint conditions (e.g., scoliosis, disc herniation, chronic neck pain).

Individuals who did not meet at least one of these criteria were excluded from the study.

### Study design

Posture assessment during supragingival scaling was performed on 10 periodontology research assistants at the Department of Periodontology, Faculty of Dentistry, Zonguldak Bülent Ecevit University. Each participant completed supragingival scaling in two patients. Each patient was divided into two groups according to the transverse quadrants (upper right and lower left quadrants = Group A and upper left and lower right quadrants = Group B), which were switched in the second patient to ensure randomization (e.g., group A was treated with a loupe in the first patient, and group B was treated with a loupe in the other patient). For the same patient, the other quadrants were treated without a loupe. A counterbalanced crossover design was implemented to minimize both carry-over and order effects. Each clinician performed supragingival scaling in two different patients. In the first patient, treatment began with the use of a loupe, followed by a non-loupe procedure. In the second patient, the sequence was reversed, starting without a loupe, and then proceeding with loupe-assisted scaling. This design ensured that the impact of the treatment order was evenly distributed across participants and conditions. Twenty quadrant pairs were treated in the upper right and lower left quadrants (Group A), while the same number of quadrant pairs were included in the study for the upper left and lower right quadrants (Group B). A total of 40 quadrant pairs were analyzed. In this study, pupil-distance adjustable flip-up loupes with an ergoprism design were used (Ergoprism Flip-up 3 × Loupe, Zumax Medical Co., Ltd., China). This type of loupe allows for flexible declination angles and individualized adjustments for interpupillary distance and working distance, enabling ergonomic optimization for each user. Posture assessment was conducted by two previously calibrated investigators (RC and MKC) in accordance with the dental ergonomic posture evaluation criteria. Each participant was allowed to adjust the patient and dental chair position according to their routine clinical working habits prior to beginning each procedure, and no standardization or external intervention was applied in this regard. No interventional periodontal treatment efficacy was evaluated in the present study.

In the evaluation of the posture periodontology research assistants, BPAI defined by Branson et al. was used, the validity and reliability of which have been demonstrated^32^. According to the BPAI, each ergonomic position category was associated with a numerical score.

One point was given for acceptable criteria, two points for compromised postures, and three points for harmful postures, indicating the extent of deviation from ideal posture. It was concluded that the higher the score, the worse the posture (Table 1)^32^.

**Table 1:** Branson’s posture assessment instrument.

Body part	Posture	1 point (Acceptable)	2 point (Compromised)	3 point (Harmful)
Hips	Stool level	Level	Not level	
Trunk	Front-back tilt	≤ 20°	> 20°, < 45°	≥ 45°
	Side-side tilt	≤ 20°	> 20°, < 45°	≥ 45°
	Rotation	≤ 20°	> 20°, < 45°	≥ 45°
Head/Neck	Front-back tilt	≤ 20°	> 20°, < 45°	≥ 45°
	Side-side tilt	≤ 20°	> 20°, < 45°	≥ 45°
	Rotation	≤ 20°	> 20°, < 45°	≥ 45°
Shoulders	Position	Relaxed	Slumped forward	
	Level	Both level	One or both elevated	
Wrist	Flexion/extension angle	≤ 15°	> 15°	

All photographs were taken using a Canon EOS 600D digital camera from three predefined positions around the dental unit (lateral, sagittal, and axial). To ensure visual consistency, all images were captured by the same individual, maintaining similar distance and positions across participants. Although the total treatment duration varied depending on the patient’s clinical needs, posture photographs were consistently captured at the 1st, 3rd, and 5th minutes of the procedure to allow for standardized comparison. The position of the patient and the dental chair during the procedure was not controlled or standardized and therefore, the dentist was allowed to adjust the patient and dental chair position according to their routine clinical working habits prior to beginning each procedure.

In this way, a total of 240 digital photographs were obtained (120 with loupe and 120 without loupe). The final score, a weighted composite score, was calculated at the first, third, and fifth minutes, based on the BPAI evaluated by the calibrated investigator. The scores from the three time points were summed and averaged, with physicians receiving a total score ranging from a minimum of 10 to a maximum of 26 points.

### Statistical analysis

The statistical analyses of the data obtained in the study were performed using SPSS 27.0 for Windows (Statistical Package for Social Sciences, Chicago, USA). The normality assumption of the variables was evaluated using the Shapiro–Wilk test (n < 50), and it was found that only the total score followed a normal distribution (*p* > 0.05).

Since the same participants were evaluated under both magnification and non-magnification conditions, a paired samples t-test was used to compare the total BPAI scores between the two conditions. For the comparison of subcomponents, the Wilcoxon signed-rank test was applied due to the non-normal distribution of the data. Spearman’s rank correlation analysis was performed to examine the pairwise associations among the BPAI subcomponents, as well as their correlations with the total BPAI score.

Additionally, intra-rater reliability was assessed by calculating the intraclass correlation coefficient (ICC) in 25% of the randomly selected samples, with repeated measurements taken two weeks apart. Two calibrated raters (RC and MKC), with 7 and 5 years of academic experience in periodontology respectively, independently evaluated anonymized digital images. The image order was randomized in both sessions, and raters were blinded to their previous assessments, to each other’s scores, and to any clinical or non-clinical cues. No reference standard was applied as BPAI scoring is subjective. This procedure adhered to the QAREL checklist^33^ for reliability studies. Furthermore, a post hoc power analysis was conducted using the method of Walter et al.^34^. Based on an observed ICC of 0.89, a null hypothesis ICC of 0.50, a sample size of 60 repeated measures, and two raters, the effect size (f) was calculated as 1.883. The resulting statistical power was 1.000 (100%), indicating that the intra-rater reliability assessment was sufficiently powered. A statistical significance level of *p* < 0.05 was considered within the 95% confidence interval.

## Results

Repeated measurements performed by the researchers (RC and MKC) showed high intra-examiner reliability, with intraclass correlation coefficients (ICCs) ranging from 0.890 to 0.974 (*p* < 0.001). This observational comparative study evaluated the effect of using and not using a loupe on the posture of periodontology research assistants during supragingival scaling. A total of 10 volunteer periodontology research assistants, 3 males and 7 females, were included in the study, and the mean age of the periodontology research assistants was 26.4 ± 1.95 years, with an average of 2.5 years of clinical experience.

The total BPAI and body component scores of the study groups are shown in Table 2. The BPAI scores during loupe use were significantly lower than those in the non-loupe condition (*p* < 0.01). The mean BPAI score during loupe use was 10.64 ± 0.531, whereas it was 13.01 ± 1.593 in the non-loupe condition. During loupe use, significantly lower BPAI scores were observed in the body components of the hip, trunk, head/neck, and shoulders (*p* < 0.05).

**Table 2:** Comparison of loupe and without loupe scores according to BPAI total and body components.

	WITHOUT LOUPE(i) Mean ± SD (Median)	LOUPE(j) Mean ± SD (Median)	Mean difference i–j (95% CI)	*p*	Effect size
Hips	1.68 ± 0.333 (1.66)	1.38 ± 0.377 (1.33)	0.495 (0.004–0.660)	**0.019**^**w**^	0.683
Trunk	3.40 ± 0.499 (3.33)	3.08 ± 0.181 (3)	0.495 (0.165–1.155)	**0.013**^**w**^	0.891
Head/neck	4.21 ± 0.741 (4.33)	3.02 ± 0.073 (3)	1.325 (0.995–1.660)	** < 0.001**^**w**^	1.00
Shoulders	2.61 ± 0.473 (2.66)	2.10 ± 0.155 (2)	0.660 (0.494–0.995)	** < 0.001**^**w**^	1.00
Wrist	1.12 ± 0.161 (1)	1.07 ± 0.135 (1)	≈0.00 (− 0.00 − 0.330)	0.351^w^	0.333
BPAI total	13.01 ± 1.593 (12.64)	10.64 ± 0.531 (10.66)	2.37 (1.61–3.13)	** < 0.001**^**t**^	1.460

^t^Paired samples t-test; ^w^Wilcoxon signed-rank test; 95% CI: 95% confidence interval. Statistical significance was considered at *p* < 0.05. Effect sizes were calculated as Cohen’s d for the paired samples t-test and as r (rank-biserial correlation) for the Wilcoxon signed-rank test. For the wrist region, the mean difference is extremely small and has been rounded for presentation clarity (≈ 0.00).

Significant values are in bold.

The correlations between the total BPAI and body component scores of the study groups are presented in Table 3. A very high positive correlation was found between the total BPAI score and head/neck body component score (r = 0.912, *p* < 0.001). High positive correlations were observed between the total BPAI score and the body component scores of the shoulder, hip, and trunk (r = 0.746, r = 0.661, and r = 0.629, respectively; *p* < *0.001*). Additionally, significant correlations were detected among the body components of the BPAI. The head/neck score showed a moderate positive correlation with the trunk score (r = 0.549, *p* < 0.001) and a high positive correlation with the shoulder score (r = 0.708, *p* < 0.001).

**Table 3:** Spearman’s rank correlation coefficient (Spearman’s rho) for BPAI total scores and body component scores with and without loupe.

	Hips	Trunk	Head/Neck	Shoulders	Wrist	BPAI total
Hips	–					
Trunk	0.216	–				
Head/Neck	0.447*	0.549**	–			
Shoulders	0.314*	0.535**	0.708**	–		
Wrist	0.046	0.011	0.241	− 0.023	–	
BPAI total	0.661**	0.629**	0.912**	0.746**	0.270	–

*Correlation is significant at the 0.05 level (2-tailed), **Correlation is significant at the 0.001 level (2-tailed).

Significant values are in bold.

**Fig. 1:**
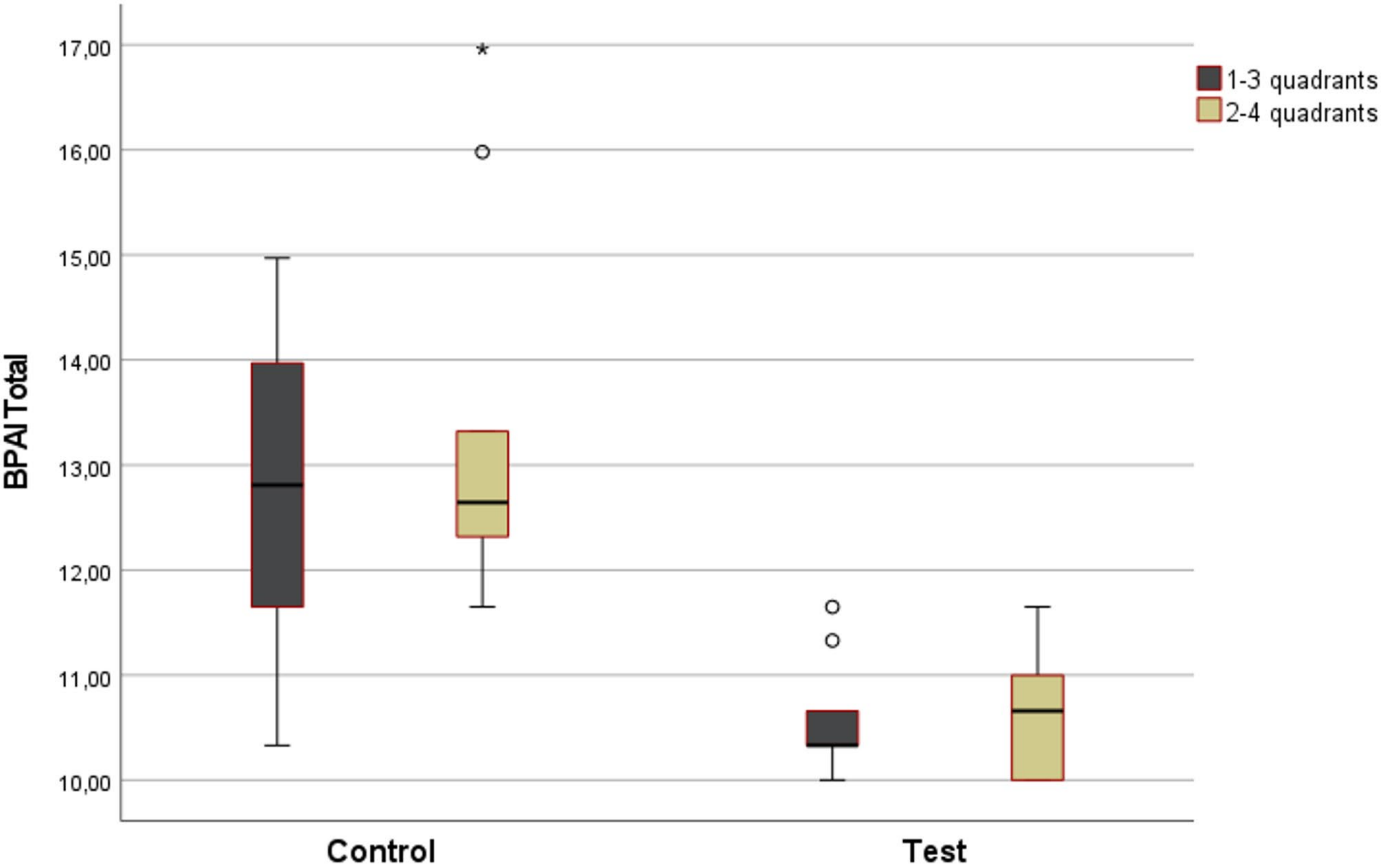
Total posture scores by quadrants in study groups.

In the control group, no significant difference was found in the total posture scores between the cross quadrants (*p* = 0.365). Similarly, in the test group, the total scores of both quadrants tended to be lower than those in the control group. However, intragroup analysis revealed no significant difference between the cross quadrants (*p* = 0.686) (Fig. 1).

## Discussion

This observational comparative study investigated the effect of loupe usage on the posture of periodontology research assistants during supragingival scaling procedures. The results were lower in the test group than in the control group in all other body assessments except for the BPAI total score and wrist component score. These findings indicate that the use of loupes has a positive effect on the posture and body components of periodontology research assistants. In relation to the primary outcome of our study, the results support the hypothesis (H1) that the use of dental loupes positively influences clinician posture during periodontal procedures. Furthermore, correlations between the BPAI total score and body components were also evaluated in the study. Accordingly, a high positive correlation was found between the head/neck and shoulder scores and the BPAI total score. This indicates that the head/neck and shoulder regions may be significant determinants in postural disorders. The meaningful correlations between the body components highlight that the components of the BPAI should be considered as a whole, rather than independently. A major strength of this study is the use of a counterbalanced crossover design involving two separate patients per clinician. By alternating the order of loupe and non-loupe conditions between patients, the study effectively mitigated potential carry-over and order effects. This methodological approach enhanced the internal validity of the findings and ensured that postural assessments were not biased by sequence-related fatigue or learning effects.

The use of BPAI in our study serves as a valuable tool to assess dentists’ posture during treatment. This tool not only helps to evaluate the impact of new equipment and devices on posture, but also demonstrates high validity and reliability in measuring dentists’ static postures at work and relates them to MSDs. Moreover, real-time clinical posture assessments can highlight subtle movement changes and thus increase the observed variability^28,32,35^.

Numerous studies have investigated the impact of magnification systems on operator posture within dental practice^36–38^. Carpentier et al. demonstrated that the use of loupes positively influences posture^39^. Although there are studies examining the overall effect of loupe usage on body posture, the number of studies specifically evaluating its impact on individual body components remains limited^36,39^. Studies that consider head-neck angular deviation have found that loupes improve neck alignment both during preclinical training and clinical procedures such as periodontal probing and diagnosis^18–20^. In the evaluation of clinician posture, the literature has primarily focused either on total body scoring or specifically on the head and neck region^38,40,41^.

To our knowledge, no previous study has investigated the relationships between individual body components and the overall posture score. In this study, a strong correlation was found between head and neck scores and the overall BPAI score, suggesting that postural deviations may initially arise in these regions. Furthermore, the significant interrelationships between body components and their collective relationship with the total BPAI score highlight the interconnected nature of posture dynamics. These findings emphasize the need to adopt a holistic approach when assessing posture.

Previous studies have evaluated the impact of different loupe systems on clinician posture during dental procedures^37,42,43^. Based on their optical design and lens configuration, loupes are commonly categorized as either Galilean or Keplerian (prismatic) systems. Galilean loupes are designed with a combination of convex and concave lenses, which together provide a clear image, a sufficient field of view, acceptable depth of field, and high image resolution^44,45^. Their simple design makes these entry-level loupes suitable for beginners and general clinical use, such as non-surgical periodontal or restorative procedures^37,46^.

Keplerian (prismatic) loupes, which consist of two or more convex lenses combined with a prism placed between them, offer a more advanced optical system compared to Galilean loupes^37^. These loupes typically provide a wider range of magnification power, most commonly between 3.5 × and 4.5 × for dental applications, although higher magnification options are also available. The inclusion of prisms extends the light path, thereby increasing the focal length and allowing for a longer working distance.

Previous studies comparing Galilean and prismatic loupe systems have generally reported that the differences between the two are not clinically significant^18,20,38^. Both systems enhance visual acuity relative to the unaided eye and contribute similarly to improvements in body posture and neck flexion^36,47,48^. To the best of our knowledge, existing research has primarily focused on the relationship between loupe use and ergonomic outcomes, particularly emphasizing the role of the declination angle in loupe selection. The enhancement in visual clarity and posture afforded by loupes plays a critical role in reducing the risk of musculoskeletal disorders among physicians, enabling the maintenance of a more natural and relaxed body position during clinical procedures^42^. Traditionally, physicians have used Through-the-Lens (TTL) loupes to achieve magnification during treatment^44^. However, these loupes typically require approximately 20° of neck flexion, forcing clinicians to adopt a non-neutral neck posture for prolonged periods. This sustained flexion leads to neck muscle fatigue and discomfort over the course of the workday. To address this ergonomic challenge, ergonomically designed loupes incorporating an ergoprism system were developed to support a neutral head and neck posture. These loupes are designed so that the eyes remain parallel to the floor, with the ears aligned over the shoulders, eliminating the need for neck flexion^37,43,49^. This posture-preserving design allows clinicians to examine patients while maintaining an anatomically neutral cervical position. Consequently, the choice of loupe system may be influenced by the clinician’s ergonomic priorities, experience, and specific clinical demands. From a design perspective, loupe systems such as Ergoprism and flip-up models are capable of minimizing head and neck angulation, offering improved declination angles and supporting better postural alignment during clinical practice.

Building upon this rationale, we also utilized pupil-distance adjustable Ergoprism Flip-up loupes in our study. Flip-up loupes are a type of loupe in which the telescopic optical barrels are connected to the frame via a hinge mechanism, allowing the lenses to be lifted upward when not in use^50^. This design offers a flexible declination angle and an adjustable interpupillary distance, making the device easily customizable for multiple users and varying working distances. Furthermore, the addition or replacement of prescription lenses to correct refractive errors is more practical in flip-up loupes compared to TTL models. In a study conducted by M. Carpentier et al., flip-up loupes were also used and were shown to help maintain clinician posture^39^. Although flip-up loupes are slightly heavier than TTL loupes due to their structural design, they allow for a better declination angle. According to Valachi, flip-up loupes offer a steeper declination angle and enable a more neutral working posture when compared to TTL loupes^48^. Similarly, Eichenberger’s^51^, study conducted in Switzerland with 69 dentists across various age groups, and the review by Perrin^52^, both reported that prismatic loupes, when compared to Galilean systems, provided higher visual acuity in dentists over the age of 40. This was attributed to their higher magnification capacity, which allows for more precise detail detection.

While the findings of this study provide meaningful insights into the research question, several limitations should be considered when interpreting the results. The single-center nature of the study, the limited sample size, and the relatively homogenous age distribution among participants may restrict the generalizability of the findings to the broader population of dental professionals. Furthermore, the study evaluated only the short-term postural effects of loupe use and did not investigate the long-term impact on musculoskeletal disorders. Postural assessments were based solely on observational methods, and no objective biomechanical measurements such as muscle activity or physical fatigue (e.g., EMG) were conducted. The position of the patient and the dental chair during the procedure was not controlled or standardized and therefore, the dentist was allowed to adjust the patient and dental chair position according to their routine clinical working habits prior to beginning each procedure. These limitations should be taken into account when interpreting the results, and future studies should consider larger sample sizes, multi-center designs, long-term follow-up, and biomechanical assessment tools. Although the BPAI includes posture categories based on estimated angular deviations, it does not provide precise continuous angular measurements. Therefore, future studies should consider incorporating more specific biomechanical assessment tools such as craniovertebral angle (CVA) analysis, which may offer greater sensitivity in detecting subtle changes, particularly in the cervical region. These limitations should be taken into account when interpreting the results, and future studies should consider larger sample sizes, multi-center designs, long-term follow-up, and biomechanical assessment tools. Moreover, the lack of an established minimal clinically important difference (MCID) for the BPAI limits the ability to determine the clinical significance of the observed improvements. Future studies should aim to define MCID thresholds for posture assessment tools to better distinguish between statistically significant findings and those that are meaningful in clinical practice.

Prior to clinical procedures, loupes were adjusted according to each clinician’s individual interpupillary distance to ensure optimal vision. However, fully customized loupes manufactured specifically for each participant were not used. Ergonomic variations stemming from this limitation were acknowledged as a potential constraint of the study. Additionally, since the majority of participants were female, it was not possible to evaluate gender-based ergonomic differences. Therefore, future research should involve larger, more balanced samples including both male and female clinicians. Moreover, further studies are warranted to investigate the effectiveness of physiotherapy-based exercise programs, stretching routines, and ergonomic training interventions in preventing and managing musculoskeletal disorders among dentists. In addition, loupe use could be evaluated across different dental specialties, and ergonomic analyses during various procedures may clarify their specific effects and support their role as protective tools for postural health. Promoting loupe use as part of routine clinical practice could raise awareness about occupational ergonomics, a perspective that may be reinforced by future multicenter studies. In this study, the use of loupes with adjustable interpupillary distance helped reduce costs and facilitated the inclusion of more participants, contributing to the study’s feasibility and scalability.

This study possesses several original features that contribute meaningfully to the existing literature. Unlike most prior research, which primarily focuses on dental students or hygienists with limited clinical experience, this investigation was conducted on postgraduate periodontology researchers actively engaged in clinical training. This distinction enhances the practical relevance of the findings and ensures stronger alignment with real-world dental practice. Moreover, the implementation of the study within an actual clinical setting, directly involving patient care, increases the external validity of the results and sets it apart from simulation-based studies by providing tangible, practice-oriented outcomes.

Another major strength of this study lies in its use of a counterbalanced crossover design, which allowed each participant to serve as their own control across both test and control conditions. This approach minimized inter-individual variability and enabled a more reliable attribution of postural differences to loupe use. Furthermore, ergonomic assessments were not limited to general postural scores but were conducted using the validated BPAI, with a detailed breakdown across specific body components (head/neck, shoulders, trunk, hips, and wrists). This comprehensive method, rarely applied in existing literature, provides a deeper understanding of the regional distribution of postural strain and musculoskeletal risk. With its well-defined participant profile, robust design, and clinically grounded execution, this study offers a high-quality and original contribution to the field of dental ergonomics.

## Conclusion

This study demonstrated that the use of dental loupes positively influences clinician posture during periodontal procedures. Significant improvements were observed particularly in the head/neck and shoulder regions, which were found to be highly correlated with the overall posture score. These findings highlight the critical role of these body components in reducing ergonomic strain. While the results suggest that loupes may provide ergonomic benefits in clinical settings, the limited sample size and short assessment period indicate that further studies are needed to validate these outcomes.

### Clinical relevance

The use of dental loupes during periodontal procedures improved clinician posture, particularly in the head/neck and shoulder regions. These results suggest that loupes can aid in ergonomic working and promote occupational health in dentistry.

## Data Availability

The datasets analyzed during the current study are available from the corresponding author on reasonable request.
